# Pathogenesis and phylogenetic analyses of canine distemper virus strain ZJ7 isolate from domestic dogs in China

**DOI:** 10.1186/1743-422X-8-520

**Published:** 2011-11-16

**Authors:** Bin Tan, Yong-Jun Wen, Feng-Xue Wang, Shu-Qin Zhang, Xiu-Dong Wang, Jia-Xin Hu, Xin-Chuan Shi, Bo-Chao Yang, Li-Zhi Chen, Shi-Peng Cheng, Hua Wu

**Affiliations:** 1 Division of Zoonoses, Institute of Special Economic Animal and Plant Sciences, State Key Laboratory of Special Economic Animal Molecular Biology, Chinese Academy of Agricultural Sciences CAAS, 15 Luming Street, Jilin 132109, China

**Keywords:** Canine distemper virus (CDV), MDCK, Genotype, Phylogenetic analysis, Pathogenesis, Virulence

## Abstract

A new isolate of canine distemper virus (CDV), named ZJ7, was isolated from lung tissues of a dog suspected with CDV infection using MDCK cells. The ZJ7 isolate induced cytopathogenic effects of syncytia in MDCK cell after six passages. In order to evaluate pathogenesis of ZJ7 strain, three CDV sero-negative dogs were intranasally inoculated with its virus suspension. All infected dogs developed clinical signs of severe bloody diarrhea, conjunctivitis, ocular discharge, nasal discharge and coughing, fever and weight loss at 21 dpi, whereas the mock group infected with DMEM were normal. The results demonstrated that CDV-ZJ7 strain isolated by MDCK cell was virulent, and the nucleotide and amino acid sequences of strain ZJ7 had no change after isolation by MDCK cell when compared with the original virus from the fresh tissues. Molecular and phylogenetic analyses for the nucleocapsid (N), phosphoprotein (P) and receptor binding haemagglutinin (H) gene of the ZJ7 isolate clearly showed it is joins to the Asia 1 group cluster of CDV strains, the predominant genotype in China.

## Introduction

Canine distemper (CD) is an acute or subacute, highly contagious disease with signs of generalized infection including respiratory disease, foot pad hyperkeratosis, central nervous system disturbance or a combination of these symptoms [1]. Its causative agent is a canine distemper virus (CDV) that is an enveloped virus particle with a diameter of 150 to 300 nm [2], belonging to the *Morbillivirus *of *Paramyxoviridae *family. CDV is actually a single-stranded negative-sense RNA virus (~15.7-kb RNA genome) and causes a highly infectious, systemic and fatal disease in the wild and domestic *Canidae *[3,4]. The virus replicates primarily in lymphatic tissues of the respiratory tract. Temporary fever and the onset of lymphopenia appear after 3 to 6 days infection [5,6]. Generally, an acute infection by CDV is associated with respiratory or gastrointestinal tract disease or both, and central nervous system [7]. The genome of CDV encodes the following virion proteins: nucleocapsid (N), phosphoprotein (P), matrix (M), fusion (F), hemagglutinin (H), and polymerase (L). H protein is responsible for viral attachment to host cell and may play a role in inducting the protective immunity as well [8]. H protein is also one of the most variable morbillivirus proteins and thus has been commonly used to assess genetic changes between CDV isolates [9]. Sequence analyses of CDV strains have been identified in diverse geographic areas and various animal species, indicating that H gene of CDV strains underwent a genetic drift related to the geographic locations of the circulating strains [10]. Dogs infected with virulence CDV strains showed obviously clinical signs of canine distemper including conjunctivitis, ocular discharge, nasal discharge, depression, coughing, diarrhea, lymphopenia, high body temperature and body weight loss [1]. All infected dogs were diagnosed with lymphopenia at 5 or 7 dpi, which is the most important clinical sign to reflect the immunosuppression [3] and may be affected by apoptosis [11]. Lymphoid depletion started in the lymph nodes and thymus at 6 dpi without necrosis [5]. However, the lymph node follicles of dogs that naturally infected with CDV have pathological findings from necrosis to lymphoid depletion [12].

An isolation of CDV strains from tissues by cell culture is difficult because the lipid-enveloped CDV is sensitive to the environment and easily inactive by heat and light [13]. However, the field isolates of CDV have been reported to be successfully replicated in macrophages of dogs and ferrets [14,15]. This attributed to many receptors on macrophages cell surface, such as the signaling lymphocyte activiation molecule (SLAM), which allows CDV strains entering the cells. Therefore, the CDV can be isolated by co-cultivation of lymphocytes from the suspected dogs and lymphocytes from mitogen-stimulated dogs [16]. Kimoto focused on the Vero cell, modified and unmodified, to isolate the CDV strains [17]. Lednicky et al. demonstrated an effective isolation of the wild-type CDV strains by MDCK, whose method is much earlier detecting the virus than others [13]. It was known that the virulence for natural host could be lost when the CDV was adapted to the cell culture [18], and so the isolation of virulence CDV from the suspected dogs is more difficult [19]. In this study, however, the virulence CDV had been isolated in MDCK cell from the infected and clinically sick dogs as early as three days after inoculation. This is may be because that MDCK cell is sensitive to the CDV filed strains and so the CDV strains can be replicated *in vitro *without selection and/or adaptation in the study. This method is an effective tool for the research of CD disease and development of CD vaccine candidate. In addition, the geographical lineage(s) of the current China CDV field strains have also been determined in this study.

## Results

### Morphology and characteristics of new CDV isolate

**Figure 1:**
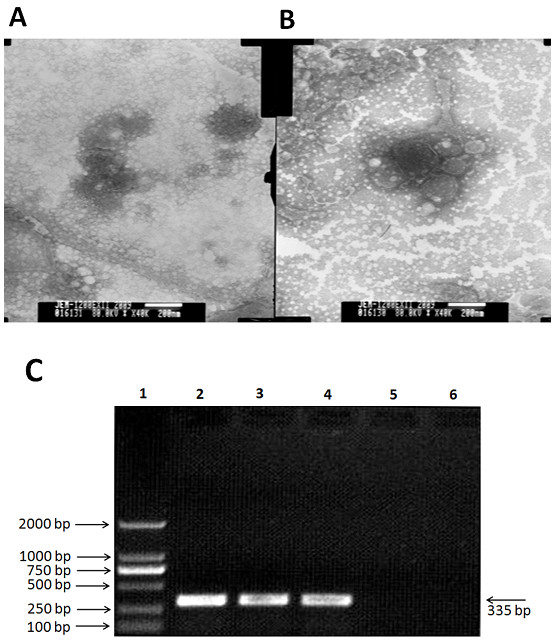
**Identification of ZJ7 isolate by morphology and molecular biology methods**. (A and B) The morphology of ZJ7 isolate under electron microscope (negative staining) and the particles of assembling viruses; (C) Electrophorogram results of RT-PCR from 3 and 6 passages, the band is 335bp, that is same size as the fragment we designed.

A wild-type CDV isolate, named ZJ7, was isolated from lung tissues of the infected dogs and examined by EM for testing the presence of virus particles. Canine distemper virus particles with typical morphology were detected by EM in stools from infected dogs (Figure 1). A virion of approximate 200-300 nm in diameter was observed in negative-stain preparations of MDCK cell inoculated with the ZJ7 isolate. After treated with FUDR, the CDV-ZJ7 virus titer was one log10 lower than the untreated virus (Table 1). After treated with ethylether, acid and heat, the CDV ZJ7 virus titer was three log10 lower than the untreated group.

**Table 1:** The tolerance of CDV-ZJ7 strain for FUDR, ether, acid and heat

Test conditions	Test group (TCID_50_)	Control group(TCID_50_)
FUDR	10^-3. 56^	10^-4. 19^
20% Ethylether, 4°C, 24 h	10^-0. 69^	10^-4. 13^
pH3.0, 37°C, 2 h	10^-0. 94^	10^-4. 06^
50°C, 30 min	10^-0. 56^	10^-4. 31^

### Cytopathic effect (CPE) on MDCK cell and IFA detection of CDV antigen

**Figure 2:**
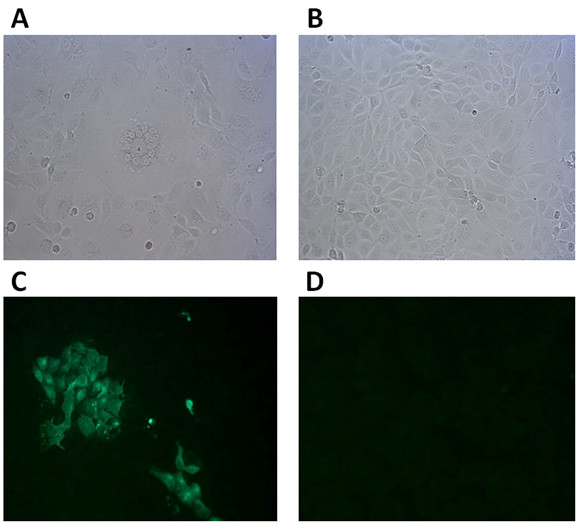
**Microphotographs and IFA results**. (A) CPE in MDCK cell with large round syncytium and multiple nuclei by CDV-ZJ7. (B) Typical CDV destruction of cell layers for mock. (C) IFA for ZJ7 isolate-infected MDCK cell and (D) for Non-infected MDCK cells. Original magnifications is 200 ×.

The homogenates from lung tissues of CDV infected samples were cultured and passaged in MDCK cells, and then one virus isolate was obtained. This virus isolate uniformly produced CPE characteristics of CDV strains after 6 passage (Figure 2A), showing many scattered, rounded, refractory cells and small syncytia. The presence of CDV isolates in MDCK cell has been confirmed by FITC-labeled CDV N protein-specific antibody. The specific green signals of FITC were detected in the infected group, whereas the mock group had no fluorescence signals (Figure 2D).

### Clinical features of the infected dogs

**Figure 3:**
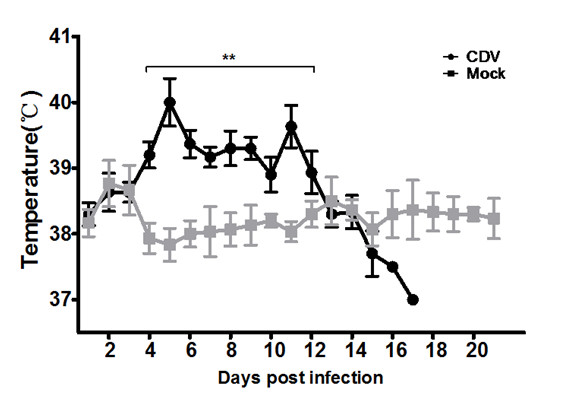
**Rectal temperatures of the three dogs after inoculation**.

**Figure 4:**
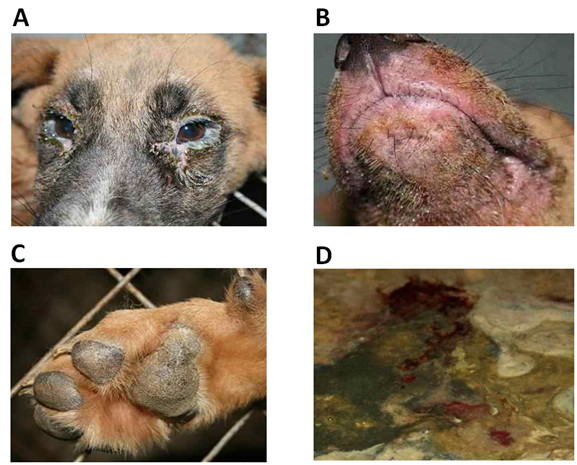
**The typical clinical signs of CDV infected dogs: (A) displayed respiratory signs with an ocular discharge from the eye; (B) exhibited clinical symptoms of full of red rashes in the face; (C) hardened footpad of the infected dogs; (D) Bloody diarrhea on the ground**.

Several clinical symptoms of CD disease have been shown in three infected dogs compared with three mock dogs that were infected with DMEM. There was a significant difference in body temperature from 4 to 12 dpi between the infected and mock groups (Figure 3). The infected dogs exhibited the elevated body temperature up to 40.5°C, and had two peaks of rectal temperature at more than 39.5°C. Ocular discharge and anorexia occurred in the infected groups between 3 and 4 dpi (Figure 4A), and they also developed tonsillitis and coughing. In addition, the infected dogs gradually became depressed and had rashes, hardened footpads and bronchitis (Figure 4B and 4C). All infected dogs developed bloody diarrhea at 4-8 dpi (Figure 4D), and died at 16-18 dpi.

### CDV detection by RT-PCR and virus recovery

CDV in the swabs from the infected dogs were detected by RT-PCR. The virus was still detected in conjunctival, nasal and swabs of one dog at 8 dpi and other dogs at 14 dpi. The virus was also re-isolated from the swabs of all infected dogs in MDCK cells, and no CDV were detected in the mock dogs By RT-PCR.

### Sequencing and phylogenic analyses for N and P genes of CDV

**Figure 5:**
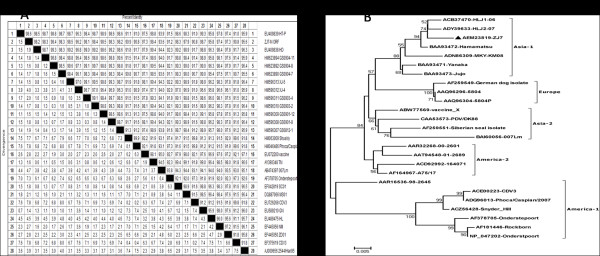
**Phylogenetic relationships between different CDV strains based on the nucleotide sequences of the coding regions of N and P gene of ZJ7 isolate compared with those of reference strains from the GeneBank**. (A) Phylogenetic tree based on the nucleotide sequences of the complete N gene. (B) Phylogenetic tree based on the nucleotide sequences of the complete P gene.

Figure 5A showed the sequence distances based on the N gene of ZJ7 isolate among 28 CDV strains obtained from the GenBank database. The homologies among ZJ7 isolate and CDV strains of TN, SC01, MS01, NM, ZD01, GN and HT-P were high with 97.3%-98.6% identity. The N gene of ZJ7 isolate showed a 93.6% identity with Japan strain 007Lm, and 91.2% identify with the classical Onderstepoort vaccine strain. There was a poor homology of the nucleotide sequence of N gene among ZJ7, CDV3 and Shuskiy that was isolated from mink in Republic of Kazakhstan. The lowest homology of 90.8% was found between ZJ7 isolate and Phoca/Caspian strains that isolated from seal. Phylogenetic analyses for P gene were also conducted in ZJ7 isolate and others CDV strains (Figure 5B). It was shown that ZJ7 isolate grouped together in one branch with Chinese strain HLJ1 and Japan strain (Hamamatsu, Jujo and Yanaka), but in a different branch with the Japan wild-type strain 007Lm and vaccine strains obviously.

### Analyses for the amino acid sequences of H gene from wild-type CDV strains

**Figure 6:**
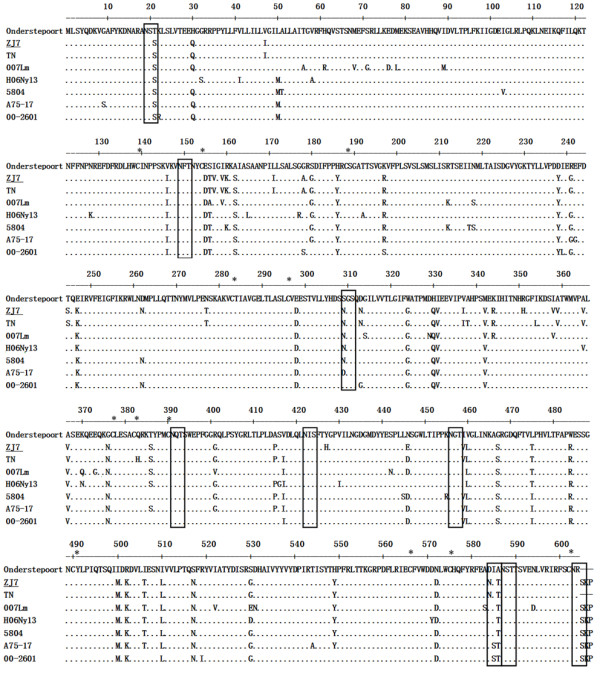
**Deduced H protein amino acid sequences and amino acid sequences**. The potential N-linked glycosylation sites are boxed. Dots (·) indicate identity. The Asterisks indicate conserved cysteine residues.

The H gene is a 1824 bp fragment, and encoding a single reading frame of 607 amino acids (Figure 6). The identity of H gene between ZJ7 and Hamamatsu strains was 98.6% in nucleotide, and 98.4% with CD TaiChung in amino acids. Consistent with other compared strains, 12 cystein residues positions on H protein of ZJ7isolate were predicted by the NetNGlyc 1.0 Server. One major hydrophobic region (amino acid 35-56) and nine potential glycosylation sites for asparagines (N)-linked glycosylation were at amino acid positions 19-21, 149-151, 309-311, 391-393, 422-424, 456-458, 584-586, 587-589 and 603-605 (Figure 6). This ZJ 7 wild-type strain had nine N-linked glycosylation sites, which was the same as the strains in the Asia 1 group, whereas the Asia 2 group only had eight sites.

## Discussion

In this study, a new ZJ7 isolate have been isolated from CV diseased dogs in Jilin province, a recent representative of CDV in China northeast, and the identification of the isolate have been confirmed by virulence investigation and molecular analysis. 100% identities of nucleotide and amino acid sequences of H and P genes have been determined between the ZJ7 strain isolated in MDCK cell and the original virus from infected fresh tissues (data not show). The results indicated that the CDV was genetically stable after isolation within a few passages and the MDCK cell was a suitable cell line to isolate CDV from fresh tissues. The ZJ7 strain isolated from several clinical cases were genetically distinct from the known vaccine strains, as previous studies of other CDV positive cases with history of CDV vaccination [19-22].

Many other studies have demonstrated the geographically distinct lineages of CDV strains by phylogenetic analysis of their H genes [19-23]. To phylogenetic analysis of ZJ7 isolate, we used the Onderstepoort (GenBank accession no. AF378705), the Snyder Hill (AF259552) and the TN (AF390348) strains as references of current China field isolates. A high degree of identity was detected among all studied China wild-type strains, which were separated from the vaccine strains from GenBank. The connected aspartic amide N glycosylation site potentially is a spotlight in H proteins between vaccine and wild strains of CDV. Usually, there are four (Onderstepoort strain) or seven (Convac strain) potential sites in the vaccine strains. However, Eight or nine sites have been detected in all wild CDV strains, of which 309 ~ 311 N-connected amide asparagine glycosylation sites are specific to CDV field strains [24]. It was consistent with pathogenic analyses of CDV ZJ7 strain in this study, where eight potential N-connected amides asparagine glycosylation including 309 ~ 311 have been confirmed in ZJ7 isolate [20]. Some studies believed that the variants from H protein glycosylation played a crucial role in the antigenic differences [21]. In addition, the predicted amino acids of ZJ7 isolate lacked 3 amino acids SKP compared with the Onderstepoort vaccine strain (Figure 6), but it had difference in 9 amino acids from another China wild-tpye TN strain.

**Figure 7:**
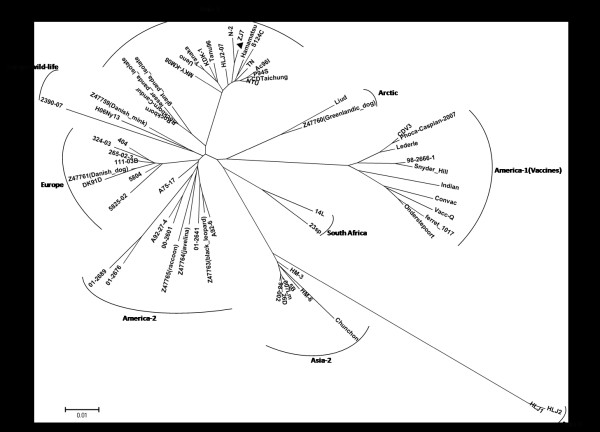
**Phylogenetic tree of ZJ7 isolate based on H protein, compared with those of reference strains from the Genebank**. GenBank accession numbers are AY466011 (CDV 98-2654), AY443350 (CDV 00-2601), and AY465925 (CDV 01-2690).

According to the phylogenetic analysis based on H gene [10,19,25], nine clades of wild-type strains have been demonstrated from different parts of the world: Aisa-1, Aisa-2, Aisa-3, Europe, Europe wild-life, America-1, America-2, South Africa and Arctic genotype CDV field strains (Figure 7). The previous study has reported that three different CDV genotypes (Asia-1, Arctic, and Asia-3) were currently circulating in China [19]. The H gene phylogenetic relationships among ZJ7 isolate and other CDV wild-type strains from GenBank have been clarified in this study. All China isolates form an Asian clade by themselves that can be clearly separated from the American, European, Arctic and vaccine virus clades. In this study, ZJ7 isolate, identified from Jilin province in 2009, displayed the highest identity to HLJ-10-6strain and can be classified into Asia-1 genotype.

## Conclusions

In this study, a CDV ZJ7 strain was successfully isolated from lung tissues of the dogs suspected with CDV infection using non-modified MDCK cells. The ZJ7 strain still induced pathogenic effects to the infected dogs after six passages, whereas the mock group infected with DMEM is normal. In addition, molecular and phylogenetic analyses of N, P, and H gene on ZJ7 isolate have clearly indicated its joins to the Asia 1 group cluster of CDV strains, the predominant genotype in China. In a word, this new CDV ZJ7 strain isolated by MDCK cell remains virulent, and its nucleotide and amino acid sequences are still conservative.

## Materials and methods

### Cell lines and culture

Cell line of MDCK (canine epithelial kidney cells) was purchased from American Type Culture Collection (ATCC) (Manassas, VA). The cells were grown routinely in polystyrene tissue culture flasks with filtercaps (Nalge Nunc International, Rochester, NY) at 37°C in a 5% humidified CO_2 _atmosphere. The culture medium was composed of Eagle's modified minimal essential medium with Earle's salts (EMEM-E) (Cellgro, Mediatech, Inc., Herndon, VA), 2.5 or 10% (as specified per application) heat-inactivated fetal bovine serum (HI-FBS, from Cellgro), antibiotics of 100 units/mL penicillin, 100 μg/mL streptomycin and 50 μg/mL gentamycin, 1 mM sodium pyruvate, 1X non-essential amino acid solution, 2 mM glutamine, and 0.075% (w/v) sodium bicarbonate.

### Viruses and clinical specimens

The CDV3 and Onderstepoort vaccine strains used in this study were maintained in the Division of Zoonoses, Institute of Special Economic Animal and Plant Sciences, China. The organs of the infected dogs were obtained from an animal hospital in Jilin province of China at 2009. The suspect of CDV infection was initially found by the doctors on the basis of the clinical signs (fever, respiratory, enteric, and hyperkeratosis signs).

### Virus isolation and titration

The virus isolation and titration were conducted as described previously [26]. Briefly, the tissue samples from the infected dogs were collected from their necropsies and then stored at -80°C. Homogenates containing 0.2 g of lung samples and 2 mL of Dulbecco's modified Eagle's medium with antibiotics were sonicated and centrifuged. A monolayer of MDCK cell was infected with the homogenates in a 24-well culture plate for isolation. ZJ7 isolate was titrated in a 10-fold dilution using a 50% tissue culture infectious dose (TCID_50_) assay [27] in a 96-well culture plate.

### Virus identification by electron microscope (EM) and immunofluorescence assay (IFA)

Before observing under EM, the MDCK cell infected with the virus isolates were harvested by freezing and thawing for three times, and a 1 mL harvested cell culture was centrifuged for 5 min at 800 ×g. The supernatant was then transferred into a new microtube and centrifuged for 10 min at 13, 400 ×g. A negative stain was prepared for transmission electron microscope observation. The observed virions were photographed and analyzed. As conventional methods [28], the isolated virus were tested by 5-fluoro deoxyuridine (FUDR), ethylether, acid and high temperature (Table 1). A 96 well microtitre plate (Costar, NY, USA) was seeded with MDCK cell in MEM with 10% NBCS, and cultivated at 37°C in 5% CO_2 _overnight until 70-80% confluence. The cultures were then inoculated with 20-fold diluted field CDV isolates at 3 passage. The uninfected cultures in left rows of the plate were taken as the negative controls. After 72 hrs incubation in 37°C, 5% CO_2 _atmosphere, the plate was fixed in 80% cold acetone/PBS, and then washed and incubated with mAbs at a 50-fold dilution. After washing with PBS, FITC-conjugated polyclonal antibody (Sigma, St. Louis, MO, USA) was added to the plate, and then followed by 1 h incubation in a 37°C humid box. After 3 times washes with PBS, a 50% glycerol in PBS was added to each well. Two infected wells were treated as a positive control to confirm viral growth. Fluorescence signal was observed using an fluorescence inverted microscope (Zeiss Axioskop-40, Germany).

### Experimental animals

Six 2-month-old female dogs, whose serology is negative to CDV, were purchased from Shifang Experiment Animal Corporation (Jiangshu, China) and raised in the isolated cages. All animal work and experimental procedures were conducted with an approval of Institutional Animal Care and Use Committee of Jinlin University, China.

### Virus infection and sample collection

A CDV ZJ7 isolate, which has been directly isolated from lung tissues of a dog showing pathological changes consistent with canine distemper at necropsy, was passaged at least five times in MDCK cell before it was used to form a viral suspension at concentration of 1.2 × 10^6 ^TCID_50 _/1 mL. Under anesthesia with protocol (Institute of Special Economic Animal and Plant Sciences, Jilin, China), the viral suspension was dropped into the right conjunctiva and nostril of three dogs using a syringe without a needle. The infection was monitored daily by clinical and neurological examination until the dogs were euthanized with Nembutal (Solabio Pharmaceutical Co. Ltd., China) at the end of the experiment. The tissue samples were obtained all infected dogs. Clinical signs and rectal temperatures of the dogs were daily recorded. Nasal, tonsilar, conjunctival, rectal and vaginal swabs, which were used for reverse transcriptase (RT)-PCR and virus reisolation, were collected at 0 (before inoculation of virus), 5, 7, 9, 10, 12, 14, 19, 21, 23 and 28 dpi. All samples for isolation were stored at -80°C until being used.

### Virus detection and recovery

A suspension containing 40 μl of nasal, tonsilar, conjunctival and vaginal swabs and antibiotics of 1000 units/mL penicillin and 1000 μg/mL streptomycin was inoculated into MDCK cell seeding in a 24-well culture plate. The cytopathogenic effect was observed by phase contrast microscopy. The presence of CDV was confirmed by reverse transcriptase-PCR with a specific CDV N gene primers: Upper: 5' GATAAAGCATGTCATTATAGTCCTAA 3' and Lower: 5'CTTGAGCTTTCGACCCTTC 3', and the expected fragment was 335 bp. Briefly, all RNA was extracted using the RNAeasy kit (QIAGEN). The extracted RNA was immediately used for RT-PCR or preserved at - 80°C before use. cDNA synthesis was performed with SuperScript II reverse transcriptase (Invitrogen) and oligodeoxy nucleotide primers by 10 mL of RNA sample and random primers as reverse transcription primer. The PCR amplification of cDNA was carried out in a 50 mL solution containing 20 mM Tris-HCl (pH 8.4), 50 mM KCl, 3 mM MgCl_2_, 0.5 mM dNTP and 200 pmol of each primer accordingly. The internal gene sequences of primers, 5mL cDNA and 2.5U EXTaq DNA polymerase (Takara) were given in the Table 1. The PCR amplification cycle was optimized as follows: 94°C 45s, 52.2°C 45s and 72°C 45s, for 35 cycles with a final extension step at 72°C for 5 min.

### Titration of VNA against CDV

For the neutralization assay, the antibody titre was measured in a 96-well culture microplate in Vero cells [29]. Quadruplicate 0.05 mL serum was diluted 3-fold serially, and a 0.05 ml CDV solution with 10 TCID_50 _of the onderstepoort strain was added to each well, and then the plate was incubated for 90 min at 37°C. Subsequently, the Vero cells at concentration of 1.2 × 10^4 ^/0.05 mL were added to the serum-virus mixtures, and incubated for 7 days at 37°C, CO_2_. The plate was examined microscopically, and the titre was expressed as the highest dilution showing 50% inhibition of cytopathic effects. Serum from a dog was vaccinated with attenuated live CDV vaccine as positive control, while virus dilution without serum was used as negative control. The titre was calculated by the Reed and Munch method [30].

### Phylogenetic analyses for nucleotide and amino acid sequences

N, P and H gene sequences of ZJ7 isolate were amplified by PCR with Pfu Turbo DNA polymerase (Stratagene), and the primers we used in Table 2. To confirm the occurrence of the target gene sequence, the amplified segments were cloned into the pMD18-T vector (TaKaRa) and sequenced at Shanghai Yingjun Biotechnological Co. Ltd (Invitrogen, Beijing, China). The nucleotide was sequenced by a commercial company and was sent to GenBank, in which the accession numbers for N, P and H were JF343964, JF343963 and JF343962 respectively. The N and P gene nucleotide were sequenced from the ZJ7 isolate and then aligned with the corresponding sequences of CDV strains using the Clustal W program in MegAlign of Lasergene 7.2 software (DNASTAR Inc. Madison, WI, USA). Then, the phylogenetic and molecular analyses were conducted using MEGA version 5 [31]. The nucleotide sequence and phylogenetic analysis were also carried out in N, P and H genes of the original homogenated tissue and ZJ7 isolate.

**Table 2:** Primers for RT-PCR and sequence analyses of CDV N, P and H genes

Gene Primer	Sequence (5'-3')	Nucleotide position
N Upper	AACAA GGCTA GGGTT CAGAC CT	80-102
N Lower	TTGTT GACTG ATGCA AGACT GGT	1688-1711
P Upper	CGACC ACCCG TTCTA TC	1779-1796
P Lower	GCGGA CTTAG GCTCT TGT	3404-3422
H Upper	CTTAG GGCTC AGGTA GTCCA	7056-7076
H Lower	ATTCA ATCGT CTGTA AGGGA	8957-8977

*Design the primers based on Onderstepoort strain of canine distemper virus

## Competing interests

None of the authors has any financial or personal relationships that could inappropriately influence or bias the content of the paper.

## Authors' contributions

BT and YJW participated in the molecular genetic studies and the sequence alignment, as well as drafted the manuscript. SQZ and BCY carried out the immunoassays. FXW participated in the sequence alignment. XDW took the animal samples and isolated the virus. JXH and XCS participated in the design of the study and performed the statistical analysis. LZC, SPC and HW conceived of the study, and participated in its design and coordination as well as helped to draft the manuscript. All authors read and approved the final manuscript.
